# HLA-Engineered iPSC-Derived Mesenchymal Stromal Cells for the Treatment of Osteoarthritis in Mice

**DOI:** 10.3390/cells15191777

**Published:** 2026-09-29

**Authors:** Ze Wei, Tian Gan, Annan Liang, Su Liu, Jing Hao, Wei Zou, Lun Li, Xuemeng Mu, Huitian Han, Wenjing Ma, Jinyi Xing, Yifan Wei, Hao Fan, Lijin Liu, Fei Liu, Zhifa Zheng, Zhihong Wu, Lina Zhao

**Affiliations:** 1 Department of Orthopedic Surgery, Peking Union Medical College Hospital, Chinese Academy of Medical Sciences & Peking Union Medical College, Beijing 100730, China; b2023001002@pumc.edu.cn (Z.W.); b2024001091@student.pumc.edu.cn (A.L.); zw1089@student.pumc.edu.cn (W.Z.); b2024001084@student.pumc.edu.cn (X.M.); hanhuitian@student.pumc.edu.cn (H.H.); b2025001181@student.pumc.edu.cn (J.X.); b2025001036@student.pumc.edu.cn (Y.W.); 2 Stem Cell Facility, Institute of Clinical Medicine, Peking Union Medical College Hospital, Chinese Academy of Medical Sciences & Peking Union Medical College, Beijing 100730, China; lius0706@bjmu.edu.cn (S.L.); haojing63@pumch.cn (J.H.); mawj@student.pumc.edu.cn (W.M.); b2025001127@student.pumc.edu.cn (H.F.); liulijin@aliyun.com (L.L.); liu_fei@bjmu.edu.cn (F.L.); zhengzhifa@pumch.cn (Z.Z.); 3 Exploratory Laboratory, Gan & Lee Pharmaceuticals, Beijing 101109, China; tian.gan@ganlee.com (T.G.); lun.li@ganlee.com (L.L.); 4 State Key Laboratory of Complex Severe and Rare Diseases, Peking Union Medical College Hospital, Chinese Academy of Medical Sciences & Peking Union Medical College, Beijing 102602, China

**Keywords:** iPSC-derived mesenchymal stromal cells, HLA engineering, engineered iMSCs, osteoarthritis

## Abstract

**Highlights:**

**What are the main findings?**

- B2M and CIITA double-knockout eliminates HLA class I and class II expression in iPSCs and iMSCs even under inflammatory conditions, while preserving MSC phenotype, proliferation capacity, and immunomodulatory function.
- In mice with OA, intra-articular administration of dKO-iMSCs reduced cartilage degeneration more effectively than wild-type iMSCs and tissue-derived MSCs, without evident systemic toxicity.

**What are the implications of the main findings?**

- HLA-engineered iPSCs provide a scalable approach for generating iMSCs with sustained loss of HLA class I and class II surface expression while preserving their therapeutic properties.
- dKO-iMSCs provide a promising platform for allogeneic OA therapy by exhibiting a less pro-inflammatory and more matrix-supportive state under inflammatory stimulation.

**Abstract:**

Osteoarthritis (OA) is one of the most prevalent degenerative joint diseases, and there is no approved pharmacological therapy that can reliably reverse cartilage degeneration or restore cartilage structure. Allogeneic mesenchymal stromal cells (MSCs) have shown therapeutic potential for OA, but inflammatory conditions can enhance HLA expression and increase allogeneic immune recognition risk. Here, we generated B2M and CIITA double-knockout induced pluripotent stem cells (dKO-iPSCs) using CRISPR/Cas9 and subsequently differentiated them into dKO-iMSCs. B2M/CIITA double-knockout did not affect iPSC pluripotency, karyotype, or teratoma formation. dKO-iMSCs retained characteristic MSC phenotypes, exhibited greater proliferative capacity than tissue-derived MSCs, and could be expanded to passage 18. Notably, HLA-ABC and HLA-DR expression remained undetectable in dKO-iMSCs even after IFN-γ stimulation. In co-culture assays with PHA-L-stimulated allogeneic PBMCs, dKO-iMSCs suppressed PBMC and T-cell proliferation as effectively as wild-type iMSCs, indicating preservation of their immunomodulatory activity. Intra-articular administration of dKO-iMSCs significantly reduced cartilage degeneration in the destabilization of the medial meniscus (DMM) mouse model of OA. The treatment showed greater therapeutic efficacy than wild-type iMSCs and tissue-derived MSCs and did not cause evident systemic organ toxicity. Single-cell RNA sequencing of BM-MSCs and dKO-iMSCs after in vitro IFN-γ stimulation revealed enhanced extracellular matrix-associated cell–cell communication and markedly reduced IL1B expression in dKO-iMSCs, indicating a less pro-inflammatory and more matrix-supportive cellular state under inflammatory conditions. Collectively, B2M/CIITA double-knockout enables sustained reduction in HLA expression while preserving the MSC phenotype and immunomodulatory activity, and enhances the therapeutic efficacy of iMSCs in the mouse OA model, providing a potential strategy for developing iPSC-derived MSCs with reduced HLA expression for allogeneic cell therapy.

## 1. Introduction

Osteoarthritis (OA) is one of the most prevalent degenerative joint diseases and represents a major cause of chronic pain, disability, and reduced quality of life worldwide [1,2,3]. The pathogenesis of OA involves a complex interplay between mechanical stress, cartilage matrix degradation, synovial inflammation, and progressive loss of articular cartilage homeostasis [4]. Current clinical management primarily focuses on alleviating pain and improving joint function through pharmacological and non-pharmacological interventions. However, there is currently no approved drug that can reverse cartilage degeneration or restore the structural and functional integrity of damaged cartilage [5,6]. Therefore, therapeutic strategies that can modulate the inflammatory joint microenvironment and promote tissue repair are highly desirable.

Mesenchymal stromal cells (MSCs) have emerged as promising candidates for cell therapy for OA owing to their immunomodulatory, anti-inflammatory, and tissue-reparative properties. MSCs are thought to exert therapeutic effects predominantly through paracrine and immunomodulatory mechanisms, including the secretion of cytokines, growth factors, extracellular vesicles, and other bioactive molecules that can modulate inflammatory responses and support tissue homeostasis, rather than replacing damaged cartilage through direct differentiation [7,8]. Clinical studies investigating MSCs for OA therapy have reported encouraging effects on pain relief, joint function, and even structural outcomes, supporting their potential as a regenerative medicine strategy [9,10]. Nevertheless, the clinical translation of tissue-derived MSCs remains constrained by several limitations. MSCs isolated from bone marrow, adipose tissue, umbilical cord, and other tissues exhibit substantial donor-to-donor variability in proliferation capacity, phenotype, and immunomodulatory activity [11,12]. In addition, the number of MSCs that can be obtained from individual donors is limited, and extensive expansion in the laboratory may lead to cellular senescence and changes in functional properties [13]. These limitations complicate the establishment of reproducible, standardized, and scalable manufacturing processes for MSC products.

Induced pluripotent stem cells (iPSCs) provide an attractive alternative source for generating MSCs and may overcome several limitations associated with tissue-derived MSCs [14,15]. Theoretically, iPSCs possess extensive self-renewal capacity and provide an unlimited source of differentiated MSCs from a well-characterized and clonally defined cell population. iPSC-derived MSCs (iMSCs) have been reported to exhibit MSC-like phenotypic and functional characteristics, such as immunomodulation, while offering advantages in terms of scalability, manufacturing consistency, and batch-to-batch reproducibility [14,15]. Importantly, the iPSC platform is highly compatible with genome engineering, allowing genetic modifications to be introduced and clonally selected before differentiation into downstream therapeutic cell types [16]. Thus, combining iPSC technology with genetic engineering provides an opportunity not only to establish a sustainable source of MSCs but also to deliberately modify their biological properties for improved therapeutic efficacy.

Although tissue-derived MSCs and iMSCs generally exhibit low immunogenicity under basal conditions, their immunological phenotype can be altered by the inflammatory microenvironment [15,17]. In fact, inflammatory cytokines such as interferon-γ (IFN-γ) can upregulate major histocompatibility complex (MHC) molecules, including human leukocyte antigen (HLA) class I and class II molecules, which may enhance recognition by the recipient immune system [18,19]. This is particularly relevant to allogeneic cell therapy, in which cells derived from an unrelated donor must persist within an immunocompetent host. Although the immunomodulatory properties of MSCs may attenuate immune responses, allogeneic MSCs are not universally immune privileged and may still be subject to immune recognition and elimination [18,20]. Therefore, reducing the immunogenicity of iMSCs while preserving their therapeutic properties is a key consideration for developing broadly applicable, “off-the-shelf” cell therapies.

Genetic engineering of the HLA system represents a potential strategy for reducing allogeneic immune recognition. HLA class I molecules play a central role in the presentation of endogenous peptides to CD8^+^ T-cells [21]. β2-microglobulin (B2M) is the invariant light chain of HLA class I molecules. B2M deficiency leads to the retention of HLA class I heavy chains within the endoplasmic reticulum (ER), thereby preventing their expression on the plasma membrane [22,23]. In parallel, class II MHC transactivator (CIITA) serves as the master transcriptional activator for HLA class II expression, binding to cis-acting elements in the promoter regions of HLA class II genes to regulate the transcription of all classical and non-classical loci [21]. Targeting CIITA provides a strategy for suppressing HLA class II expression [24,25]. Studies of HLA modification in MSCs have predominantly focused on B2M disruption [22,26,27]. However, B2M disruption alone addresses HLA class I but does not prevent the induction of HLA class II under inflammatory conditions. In particular, IFN-γ can upregulate HLA class I and induce HLA class II expression in MSCs, potentially increasing their HLA-mediated immune visibility within an inflammatory microenvironment [28]. Although combined B2M and CIITA disruption has been explored in several cell types [23], its application to MSCs, particularly iPSC-derived MSCs, remains comparatively less explored. Combined disruption of B2M and CIITA may consequently reduce both HLA class I and class II-mediated allogeneic immune recognition.

In this study, we generated B2M and CIITA double-knockout iPSCs (dKO-iPSCs) and subsequently differentiated them into double-knockout iMSCs (dKO-iMSCs). We characterized dKO-iMSCs to determine whether B2M and CIITA disruption reduces HLA expression while preserving the immunomodulatory and therapeutic properties of MSCs. The therapeutic potential of these cells was further evaluated in mouse OA models, and single-cell transcriptomics was employed to explore their mechanisms of action. By integrating HLA-targeted genome engineering with the scalable iPSC platform, this study aims to establish a potential strategy for generating more immunologically compatible iMSCs and to provide a foundation for the development of allogeneic cell-based therapies for OA and other regenerative medicine applications.

## 2. Materials and Methods

### 2.1. Cell Culture

Human iPSCs (Cellapy, Beijing, China) were maintained on Matrigel (Corning, Corning, NY, USA)-coated plates in PSCeasyIImedium (Cellapy, Beijing, China) at 37 °C with 5% CO_2_, and passaged when they reached 70–80% confluency using Versene (ThermoFisher, Waltham, MA, USA). Human BM-MSCs were purchased from Harmocell (Beijing, China) and cultured in α-MEM medium (Gibco, New York, NY, USA) supplemented with 10% FBS at 37 °C with 5% CO_2_. All cells were routinely tested for mycoplasma contamination and confirmed negative.

### 2.2. Generation of B2M/CIITA Double-Knockout iPSCs

The B2M and CIITA genes were disrupted by CRISPR/Cas9. sgRNAs targeting exon 2 of B2M (5′-CGTGAGTAAACCTGAATCTT-3′) and exon 3 of CIITA (5′-GATATTGGCATAAGCCTCCC-3′) [23] were synthesized and purified by GenScript Inc. (Nanjing, China). Before electroporation, WT-iPSCs were dissociated into single cells using Accutase (ThermoFisher, Waltham, MA, USA). Cas9 protein (IDT, Newark, NJ, USA) was complexed with the corresponding sgRNAs to generate ribonucleoprotein (RNP) complexes incubated for 15 min at room temperature. The RNP complexes were delivered into WT-iPSCs by electroporation using the P3 Primary Cell 4D-Nucleofector Kit and a 4D-Nucleofector system (Lonza, Basel, Switzerland) with program CA-137. Following electroporation, cells were recovered and maintained under standard iPSC culture conditions. After one passage, cells were treated with IFN-γ (20 ng/mL, PeproTech, Cranbury, NJ, USA) for 48 h to induce HLA expression. HLA-ABC and HLA-DR double-negative cells were subsequently isolated as single cells by fluorescence-activated cell sorting and expanded to establish individual clones. Genomic DNA from individual clones was subjected to Sanger sequencing of the B2M and CIITA target loci. Clones carrying biallelic frameshift mutations in both genes were selected and designated as double-knockout iPSCs (dKO-iPSCs). Karyotype analysis was performed by G-banding.

### 2.3. Immunofluorescence

Cells were fixed, permeabilized, and blocked before incubation with primary antibodies against OCT4 (#MA5-14845, Invitrogen, Waltham, MA, USA) and TRA-1-60 (#MAB4360, Invitrogen, Waltham, MA, USA) overnight at 4 °C. After washing, cells were incubated with fluorescently conjugated secondary antibodies (#A-11012 and #SA5-10385, Invitrogen, Waltham, MA, USA), followed by counterstaining with DAPI. Images were acquired using a Nikon confocal microscope (Nikon Corporation, Tokyo, Japan).

### 2.4. Flow Cytometry

For HLA induction, cells were treated with IFN-γ (20 ng/mL; Cranbury, NJ, PeproTech) for 48 h and subsequently harvested, washed, and stained with fluorophore-conjugated antibodies against HLA-ABC and HLA-DR (#311410 and #311404, BioLegend, San Diego, CA, USA). For MSC immunophenotyping, cells maintained under standard culture conditions were harvested and stained with fluorophore-conjugated antibodies against CD73 (#344004, BioLegend, San Diego, CA, USA), CD90 (#328110, BioLegend, San Diego, CA, USA), CD105 (#560839, BD Biosciences, Franklin Lakes, NJ, USA), CD34 (#B49202, Beckman Coulter, Brea, CA, USA), CD45 (#384406, BioLegend, San Diego, CA, USA), CD14 (#A07764, Beckman Coulter, Brea, CA, USA), and CD19 (#A07771, Beckman Coulter, Brea, CA, USA). All antibodies were incubated with cells for 30 min at 4 °C. After washing, cells were analyzed using a flow cytometer. Appropriate unstained and single-stained controls were included for flow cytometric analysis.

### 2.5. Teratoma Formation

For teratoma formation assays, 2 × 10^6^ WT-iPSCs or dKO-iPSCs were resuspended and mixed with Matrigel (Corning, Corning, NY, USA) before subcutaneous injection into 6-week-old NOD/SCID mice (Vital River, Beijing, China). Teratomas were harvested 6 weeks after injection, fixed in 4% paraformaldehyde, embedded in paraffin, and sectioned for histological analysis. Hematoxylin and eosin staining was performed to assess tissue differentiation. The formation of tissues representing all three germ layers, including ectoderm, mesoderm, and endoderm, was evaluated by histological examination.

### 2.6. Differentiation of iPSCs into iMSCs

WT-iPSCs and dKO-iPSCs were differentiated into iMSCs by adherent differentiation. Briefly, iPSCs were cultured on Matrigel-coated plates in PSCeasyIImedium and switched to differentiation medium A on day 0. The differentiation medium A consisted of Dulbecco’s modified Eagle’s medium (DMEM, Gibco, New York, NY, USA) supplemented with 10% KnockOut™ Serum Replacement (ThermoFisher, Waltham, MA, USA), 50 μM L-ascorbic acid (ThermoFisher, Waltham, MA, USA), 2 mM L-glutamine (ThermoFisher, Waltham, MA, USA), 0.1 mM non-essential amino acids (ThermoFisher, Waltham, MA, USA), and 5 ng/mL basic fibroblast growth factor (bFGF, MCE, Monmouth Junction, NJ, USA). Cells were maintained in differentiation medium for 14 days. Following differentiation, cells were passaged onto 0.1% gelatin-coated plates and designated as passage 1 (P1). Cells were subsequently cultured in differentiation medium B, consisting of DMEM supplemented with 10% FBS, 5 ng/mL bFGF, and 10 ng/mL epidermal growth factor (EGF, MCE, Monmouth Junction, NJ, USA), until day 21, and then transferred to maintenance medium, consisting of α-MEM supplemented with 10% FBS, until day 28. At day 28, the cells were designated as passage 3 (P3) iMSCs. WT-iMSCs and dKO-iMSCs were subsequently expanded and used for the indicated experiments.

### 2.7. Multilineage Differentiation Potential of iMSCs

Osteogenic, adipogenic, and chondrogenic differentiation was induced using commercial differentiation media (Cyagen, Guangzhou, China) according to the manufacturer’s instructions. After 21 days of osteogenic induction, mineralized nodules were tested by Alizarin Red S staining. After 14 days of adipogenic induction, intracellular lipid accumulation was assessed by Oil Red O staining. For chondrogenic differentiation, pellet cultures were maintained for 28 days and glycosaminoglycan deposition was visualized by Alcian blue staining.

### 2.8. Proliferation and Long-Term Expansion of MSCs

For growth kinetics analysis, WT-iMSCs and dKO-iMSCs were seeded at a fixed density of 8 × 10^4^ cells per passage and cultured until reaching approximately 70–80% confluence. Population doubling (PD) for each passage was calculated as:

PD = log_2_(N_h_/N_s_) (1)

where N_h_ represents the number of cells harvested and N_s_ represents the number of cells initially seeded. Cumulative population doubling (CPD) was calculated as the sum of PD values across successive passages. Population doubling time (PDT) was calculated as:

PDT = T/PD(2)

where T represents the duration of each culture period between seeding and harvest.

For long-term expansion analysis, WT-iMSCs and dKO-iMSCs were passaged from P3 to P18 under the same culture conditions. BM-MSCs were passaged from P3 to P12 under the same culture conditions. The corresponding PD and PDT values were calculated based on the harvested cell number, initial seeding number, and culture duration. Growth kinetics and long-term proliferative capacity were compared among WT-iMSCs, dKO-iMSCs, and BM-MSCs across the indicated passages.

### 2.9. MSCs and PBMCs Co-Culture Assay

Peripheral blood mononuclear cells (PBMCs) were purchased from Vital River (Beijing, China). For the PBMC proliferation assay, PBMCs were first labeled with CellTrace CFSE (#C34554, ThermoFisher, Waltham, MA, USA) according to the manufacturer’s instructions. CFSE-labeled PBMCs were then co-cultured with WT-iMSCs or dKO-iMSCs at a PBMC ratio of 5:1 in the presence of PHA-L (2.5 μg/mL), culturing in RPMI-1640 medium (Gibco, New York, NY, USA) supplemented with 10% FBS. A total of 40,000 WT-iMSCs or dKO-iMSCs were seeded per well in 24-well plates. PHA-L stimulation and iMSC co-culture were initiated simultaneously and maintained for 72 h at 37 °C with 5% CO_2_. Following co-culture, cells were harvested and stained with fluorophore-conjugated antibodies against CD4 and CD8 (#344632 and #344722, BioLegend, San Diego, CA, USA). Flow cytometry was used to identify CD4^+^ and CD8^+^ T-cell populations and to evaluate their proliferation based on CFSE dilution. Appropriate unstained and single-stained controls were included for flow cytometric analysis.

### 2.10. In Vivo Mouse OA Model and Therapeutic Evaluation

All animal care and experimental procedures were performed in accordance with the National Institutes of Health (NIH) Guide for the Care and Use of Laboratory Animals and approved by the Institutional Ethics Committee of Peking Union Medical College Hospital (XHDW-2025-202). Osteoarthritis was induced in 8-week-old male C57BL/6 mice by destabilization of the medial meniscus (DMM). Mice were randomly assigned to five groups (*n* = 5 per group). Briefly, mice were anesthetized, and the surgical site was shaved and disinfected before arthrotomy. The medial meniscotibial ligament was transected, and a portion of the medial meniscus was removed to induce post-traumatic osteoarthritis. The sham group underwent arthrotomy without disruption of the medial meniscus. At 3 and 5 weeks after DMM surgery, mice received intra-articular injections of 10 μL PBS, BM-MSCs, WT-iMSCs, or dKO-iMSCs (2 × 10^4^ cells per injection) into the right hindlimb knee joint using a 30-gauge needle. Mice were euthanized at week 7, and knee joints were collected for histological evaluation.

### 2.11. Histological Analysis

Knee joints were fixed, decalcified, embedded in paraffin, and sectioned at 4 μm. Sections were stained with hematoxylin and eosin and Safranin O-Fast Green to evaluate cartilage morphology, proteoglycan loss, and structural damage. Cartilage degeneration was independently assessed by two observers blinded to the treatment groups using the Osteoarthritis Research Society International (OARSI) scoring system [29]. Total OARSI scores as well as tibial and femoral compartment scores were recorded. Heart, liver, spleen, lungs, and kidneys were weighed at the endpoint, and major organs were processed for hematoxylin and eosin staining to evaluate gross and histological toxicity.

### 2.12. Single-Cell RNA Sequencing and Data Analysis

The same preparations of BM-MSCs and dKO-iMSCs used for intra-articular injection were additionally stimulated with IFN-γ (20 ng/mL) for 48 h, and single-cell suspensions were processed on the 10x Genomics Chromium platform (3′ scRNA-seq). Single-cell RNA-sequencing raw FASTQ files were processed by Cell Ranger (v8.0.1) for read alignment, cell barcode demultiplexing, unique molecular identifier (UMI) counting, and gene-expression matrix generation. The resulting count matrix was imported into R (v4.4.0) for downstream analysis using the Seurat package (v5.1.0). Cells expressing fewer than 200 or more than 6000 genes, or with more than 10% mitochondrial genes, were excluded. The filtered expression matrix was normalized, and highly variable genes across cells were identified. Then, gene expression values were scaled to remove unwanted variation. PCA was carried out on variable genes for linear dimensional reduction. Elbow plot and JackStraw analysis were used to determine statistically significant principal components, which were used as input data for non-linear dimensional reduction (UMAP). Cells were clustered with the Louvain community-detection algorithm. Differentially expressed marker genes of each cell cluster were calculated. According to well-known cell-specific marker genes, each cluster was manually annotated to corresponding cell types. Cells with a UMI count > 0 for a specific gene were regarded as positive-expressing cells for that gene. Differential expression analysis between the dKO-iMSC and BM-MSC groups was performed within each subcluster with FindMarkers using the two-sided Wilcoxon rank-sum test with Bonferroni correction; genes with a log_2_ fold change ≥ 1.5 and *p* ≤ 0.05 were considered differentially expressed. Differentially expressed genes were subjected to Gene Ontology (GO) functional annotation and Kyoto Encyclopedia of Genes and Genomes (KEGG) pathway enrichment analysis using the clusterProfiler package. To explore intercellular signaling crosstalk, cell–cell communication among the eight subclusters was predicted independently for dKO-iMSC and BM-MSC samples with CellChat (version 2.1.2) with the CellChatDB human ligand–receptor database. For each signaling pathway, interaction weight was calculated as the cumulative sum of all interaction probabilities, and signaling patterns were subsequently compared between the dKO-iMSC and BM-MSC groups.

### 2.13. Quantification and Statistical Analysis

Statistical analyses were performed using GraphPad Prism v10.2.3 (GraphPad Software, Boston, MA, USA). Statistical significance was assessed using two-tailed Student’s *t*-tests or one-way or two-way ANOVA, as appropriate. For ANOVA, appropriate post hoc multiple-comparison tests were applied where indicated. A *p* value < 0.05 was considered statistically significant. Statistical significance is indicated as follows: * *p* < 0.05, ** *p* < 0.01, *** *p* < 0.001, and **** *p* < 0.0001. The definition of *n* is provided in the corresponding figure legends, representing either independent differentiations of iPSC-derived iMSC or individual animals. The definitions of data center and error bars are also specified in the figure legends.

## 3. Results

### 3.1. Establishment and Characterization of Double-Knockout iPSC Single-Cell Clone

**Figure 1:**
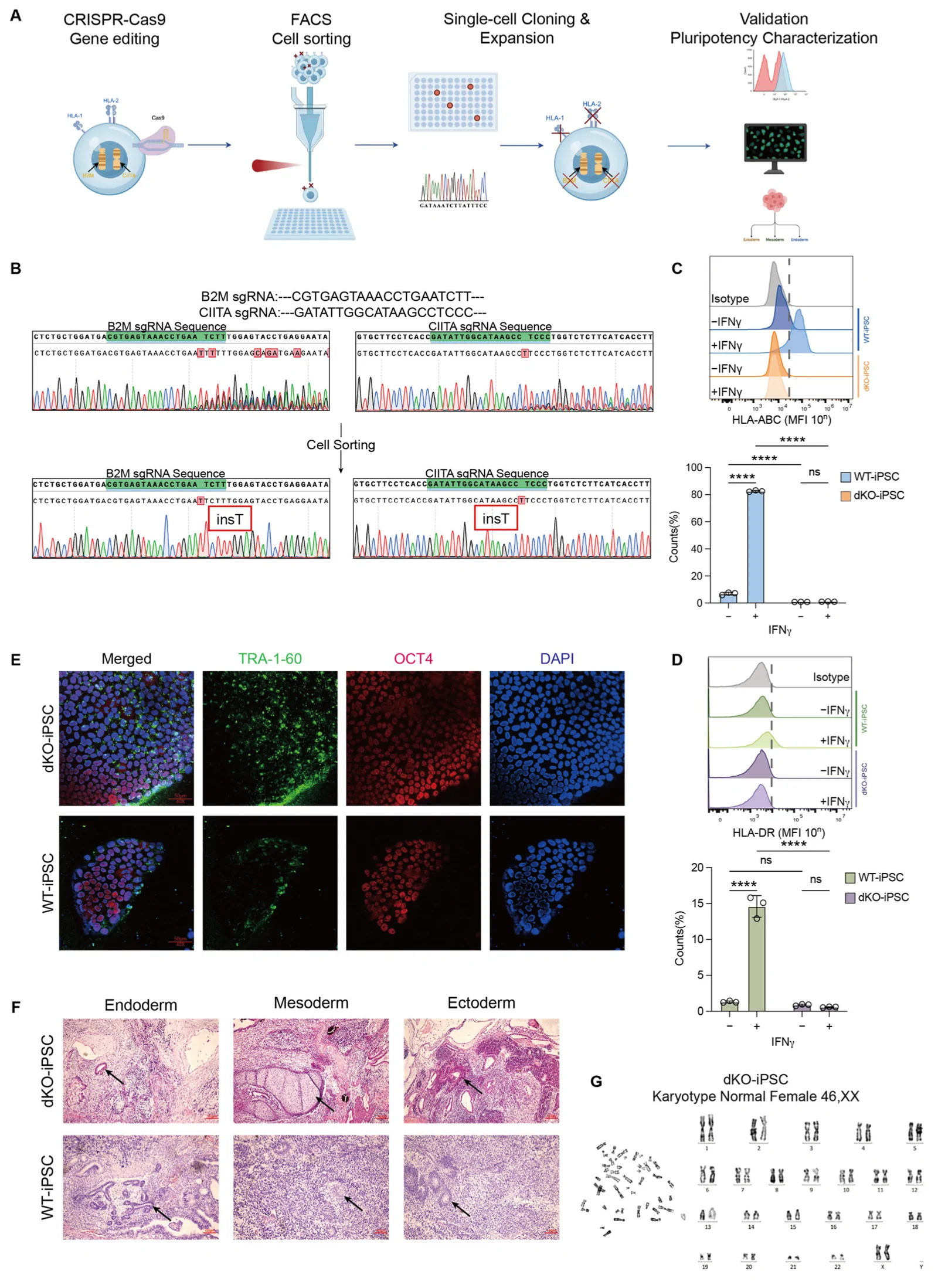
Generation and characterization of B2M/CIITA double-knockout iPSCs. (**A**) Schematic of the CRISPR/Cas9 gene-editing workflow, including RNP delivery, FACS sorting of HLA-ABC/HLA-DR double-negative single cells, single-cell cloning and expansion, and validation of pluripotency. (**B**) Sanger sequencing chromatograms of the B2M and CIITA target loci in edited clones. The sgRNA sequences are shown above the chromatograms, and red letters indicate sequence alterations induced by non-homologous end joining (NHEJ). Mixed peaks observed before clonal selection were resolved into clear, single peaks following clonal selection. The selected clone carries a single-thymine insertion in B2M and a single-thymine insertion in CIITA. (C, D) Flow cytometric analysis of HLA-ABC (**C**) and HLA-DR (**D**) surface expression in WT-iPSCs and dKO-iPSCs with or without IFN-γ stimulation (20 ng/mL, 48 h). Bar graphs show the percentage of HLA-positive cells. Statistical analysis was performed using two-way ANOVA. (**E**) Immunofluorescence staining of TRA-1-60 (green) and OCT4 (red) in WT-iPSCs and dKO-iPSCs. DAPI (blue) indicates nuclei. (**F**) Representative H&E-stained teratoma sections showing differentiation into ectoderm (neural epithelium), mesoderm (cartilage), and endoderm (glandular epithelium) in WT-iPSCs and dKO-iPSCs. Arrows indicate representative differentiated tissues. (**G**) G-banded karyotype of dKO-iPSCs showing a normal female karyotype (46, XX). Data are presented as mean ± SD (*n* = 3). ns, not significant, **** *p* < 0.0001.

In this study, we utilized the CRISPR/Cas9 system to disrupt the second exon of the B2M gene and the third exon of the CIITA gene, thereby depleting the expression of HLA class I and II molecules in iPSCs, respectively [23]. Subsequently, HLA-negative single cells were enriched and isolated via fluorescence-activated cell sorting (FACS), and clones with biallelic knockout of both genes were further verified to generate the stable B2M/CIITA double-knockout iPSCs (dKO-iPSCs) (Figure 1A). Sanger sequencing analysis verified that CRISPR/Cas9 editing induced non-homologous end joining (NHEJ) events at the target loci of B2M and CIITA (Figure 1B). Following single-cell cloning, we obtained a monoclonal cell line carrying a single-thymine insertion within the sgRNA target sequence of B2M exon 2 and a single-thymine insertion within the sgRNA target sequence of CIITA exon 3. Both insertions resulted in frameshift mutations and were predicted to generate truncated proteins (Figure 1B). Consistent with the genotyping results, flow cytometry was performed to evaluate HLA expression at the protein level; dKO-iPSCs exhibited complete depletion of cell surface HLA-ABC (HLA class I) and HLA-DR (HLA class II) proteins. In contrast, unmodified iPSCs (WT-iPSCs) showed expression of HLA-ABC and HLA-DR (Figure 1C,D). To further confirm the functional knockout under inflammatory conditions, cells were stimulated with interferon-γ (IFN-γ) to induce HLA expression. IFN-γ treatment significantly upregulated HLA class I expression in WT-iPSCs, increasing the positive population from 6.0% to 82.0%, and moderately induced HLA class II expression from 1.0% to 15.0%. However, no HLA-ABC or HLA-DR expression was detected on dKO-iPSCs even after IFN-γ stimulation, providing definitive evidence that the dual-gene knockout was complete and irreversible (Figure 1C,D).

The dKO-iPSC clones exhibited typical pluripotent stem cell colony morphology, which was identical to the morphology of WT-iPSCs. Immunofluorescence staining verified that dKO-iPSCs maintained high expression levels of the cell surface pluripotency marker TRA-1-60 and the nuclear pluripotency marker OCT4 (Figure 1E). To assess the in vivo pluripotency of dKO-iPSCs, we performed a teratoma formation assay in immunodeficient mice. Histological analysis revealed that dKO-iPSCs could effectively differentiate into tissues representative of all three germ layers, including neural epithelium (ectoderm), cartilage (mesoderm) and glandular epithelium (endoderm) (Figure 1F). In addition, karyotype analysis confirmed that dKO-iPSCs possessed a normal karyotype with no numerical or structural chromosomal aberrations (Figure 1G). These data collectively demonstrate that CRISPR-Cas9-mediated B2M and CIITA double knockout does not compromise the pluripotency or genomic integrity of human iPSCs.

### 3.2. Generation and Characterization of iMSCs Derived from Double-Knockout iPSCs

**Figure 2:**
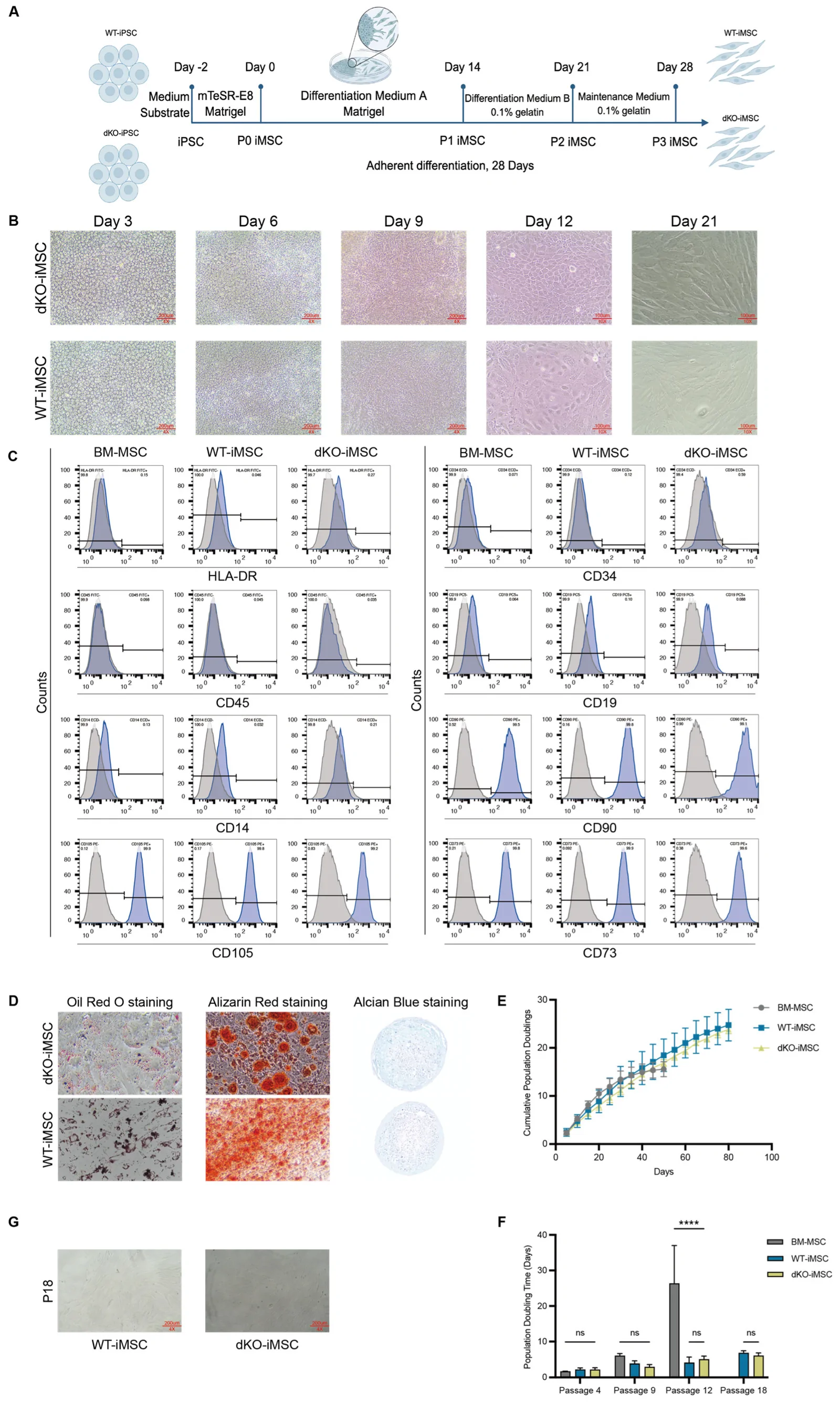
Differentiation and characterization of iMSCs derived from double-knockout iPSCs. (**A**) Schematic timeline of adherent iMSC differentiation. iPSCs were switched to differentiation medium A on day 0 and maintained for 14 days, followed by passage onto 0.1% gelatin-coated plates as P1. Cells were subsequently cultured in differentiation medium B and maintenance medium until day 28. P3 and later passages iMSCs were used for subsequent experiments. (**B**) Representative bright-field images of WT-iMSCs and dKO-iMSCs during differentiation at days 3, 6, 9, 12, and 21. Scale bars, 200 μm at 4× magnification and 100 μm at 10× magnification. (**C**) Flow cytometry of MSC markers (CD73, CD90, CD105) and negative markers (HLA-DR, CD34, CD45, CD14, CD19) in BM-MSCs, WT-iMSCs, and dKO-iMSCs. (**D**) Oil Red O, Alizarin Red S, and Alcian blue staining of WT-iMSCs and dKO-iMSCs after adipogenic, osteogenic, and chondrogenic induction, respectively. (**E**) Cumulative population doublings of BM-MSCs, WT-iMSCs, and dKO-iMSCs over successive passages. (**F**) Population doubling time at passages 6, 9, 12, and 18. BM-MSCs showed a marked growth arrest at passage 12. Statistical analysis was performed using two-way ANOVA. (**G**) Morphology of WT-iMSCs and dKO-iMSCs at passage 18. Data are presented as mean ± SD (*n* = 3). ns, not significant, **** *p* < 0.0001.

Following the generation and validation of dKO-iPSC clones, WT-iPSCs and dKO-iPSCs were differentiated into MSCs using an adherent induction protocol to assess whether B2M/CIITA double-knockout affected the differentiation process, cellular phenotype, or basic biological properties of the resulting iMSCs (Figure 2A). Both groups exhibited similar differentiation kinetics and morphological changes throughout the induction process. On day 6 of differentiation, spindle-shaped cells emerged from the edges of the iPSC colonies. Between days 9–12, the cells gradually lost the characteristic round compact colony morphology and high nuclear-to-cytoplasmic ratio of undifferentiated iPSCs. After the first passage, the majority of cells had acquired a typical mesenchymal morphology, characterized by a fibroblast-like appearance, radial arrangement, and strong adherence to the culture surface (Figure 2A,B). Microscopic examination revealed no apparent differences between WT-iMSCs and dKO-iMSCs in cell morphology, size, or growth pattern. Both populations displayed a relatively homogeneous fibroblast-like morphology without obvious morphological abnormalities (Figure 2B). These findings indicate that B2M/CIITA double-knockout did not visibly alter the morphological characteristics of iMSC differentiation from iPSCs.

To further validate the cellular phenotype at the molecular level, we analyzed the surface marker profile of the resulting MSCs by flow cytometry. Both WT-iMSCs and dKO-iMSCs exhibited a phenotype consistent with the minimal criteria established by the International Society for Cell & Gene Therapy (ISCT). Specifically, both cell populations showed high expression of MSC markers CD73, CD90, and CD105, with positive rates exceeding 98%. In contrast, less than 1% of cells in either population expressed the hematopoietic markers CD34 and CD45, the monocyte marker CD14, the B-cell marker CD19, or the class II HLA molecule HLA-DR (Figure 2C). These results indicate that double-knockout did not alter the characteristic phenotype of dKO-iMSCs, with dKO-iMSCs exhibiting a surface marker profile comparable to that of WT-iMSCs.

Trilineage differentiation assays further confirmed the multilineage differentiation potential of WT-iMSCs and dKO-iMSCs. Following 21 days of osteogenic induction, Alizarin Red S staining revealed abundant orange-red mineralized nodules in both cell populations. After 14 days of adipogenic induction, Oil Red O staining demonstrated extensive accumulation of red lipid droplets within the cytoplasm of both WT-iMSCs and dKO-iMSCs. Following 28 days of chondrogenic induction, Alcian blue staining showed prominent blue glycosaminoglycan deposition within the chondrospheres formed by both cell populations (Figure 2D). These results indicate that double-knockout did not markedly impair the osteogenic, adipogenic, or chondrogenic differentiation potential of iPSC-derived MSCs.

The growth kinetics of WT-iMSCs and dKO-iMSCs were assessed by cell population doubling analysis. No significant difference in proliferation rate was observed between the two groups, and both cell populations exhibited comparable growth kinetics in vitro (Figure 2E). To further evaluate their long-term expansion capacity, human bone marrow-derived MSCs (BM-MSCs) were included as a primary tissue-derived MSC, and cell growth was monitored across successive passages. Before P6, BM-MSCs exhibited a higher proliferation rate than both WT-iMSCs and dKO-iMSCs. Between P6 and P8, the proliferation rates of the three cell populations gradually converged, with no significant differences observed among the groups. From P8 onward, BM-MSCs showed a marked decline in proliferation and progressive growth arrest, whereas both WT-iMSCs and dKO-iMSCs maintained relatively stable proliferative activity and could be continuously expanded to P18 without evident growth retardation. No significant difference in population doubling time was observed between WT-iMSCs and dKO-iMSCs across the examined passages (Figure 2F). Morphological examination during long-term culture showed that dKO-iMSCs retained their characteristic fibroblast-like morphology through P18. No apparent enlargement of cell size or increase in cytoplasmic granularity, which are commonly associated with cellular senescence, was observed (Figure 2G).

These findings demonstrate that B2M and CIITA knockout does not compromise the fundamental cellular characteristics or proliferative capacity of the resulting iMSCs, providing a suitable cellular basis for subsequent evaluation of their immunomodulatory activity and therapeutic potential in OA.

### 3.3. Double Knockout Eliminates HLA Expression Without Compromising Immunomodulatory Function in iMSCs

Following the induction and characterization of iMSCs, we next evaluated HLA expression and in vitro immunomodulatory properties between WT-iMSCs and dKO-iMSCs.

**Figure 3:**
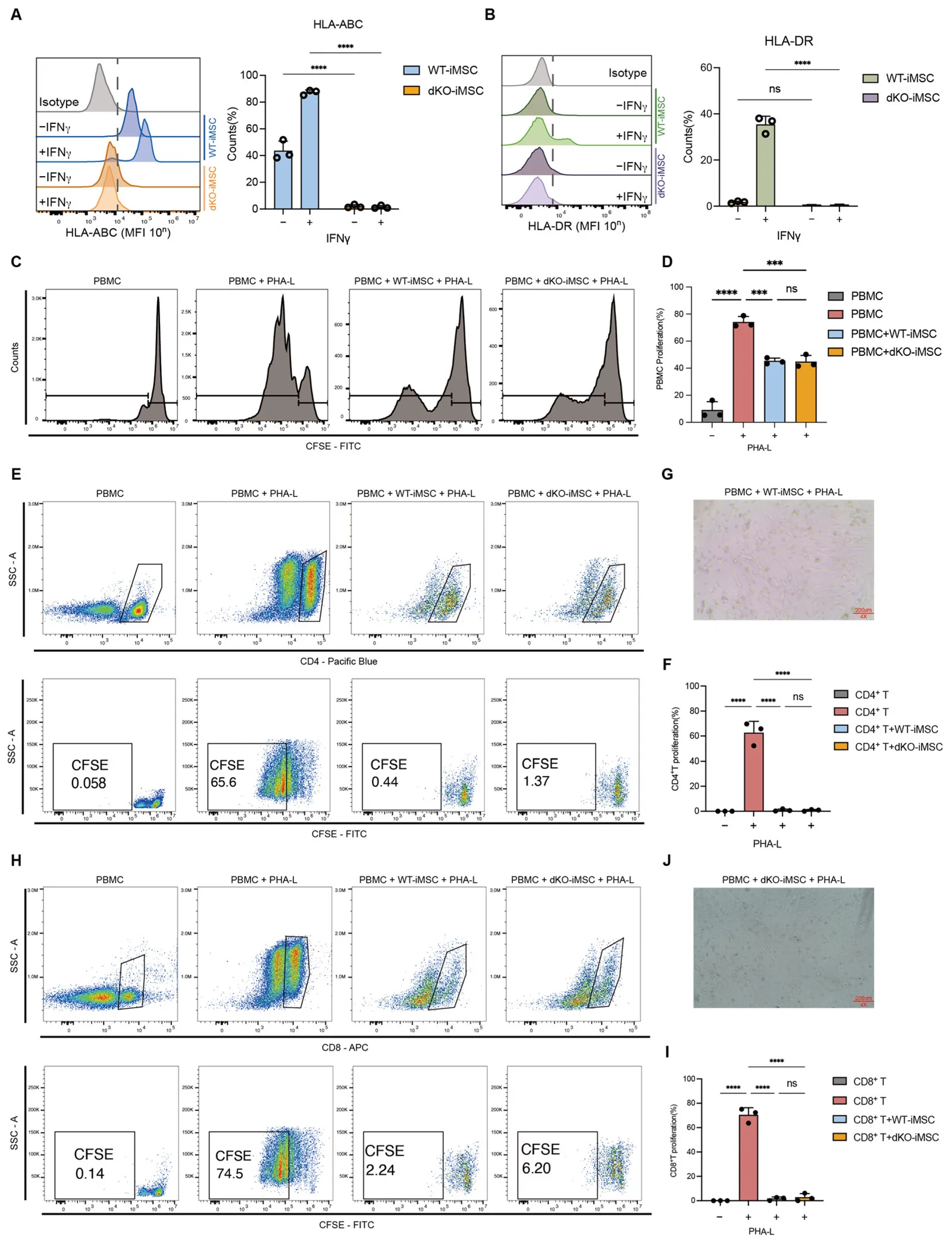
HLA silencing and preserved immunomodulatory function of dKO-iMSCs. (**A**,**B**) Flow cytometric analysis of HLA-ABC (**A**) and HLA-DR (**B**) surface expression in WT-iMSCs and dKO-iMSCs with or without IFN-γ stimulation (20 ng/mL, 48 h). Bar graphs show the percentage of HLA-positive cells. Statistical analysis was performed using two-way ANOVA. (**C**,**D**) CFSE dilution histograms (**C**) and quantification (**D**) of PBMC proliferation in unstimulated PBMCs, PHA-L-stimulated PBMCs, and PHA-L-stimulated PBMCs co-cultured with WT-iMSCs or dKO-iMSCs for 72 h. Statistical analysis was performed using one-way ANOVA. (**E**,**H**) Representative flow cytometry plots showing CD4^+^ (**E**) and CD8^+^ (**H**) T-cell gating (top row) and CFSE dilution profiles of the corresponding gated T-cell populations (bottom row). (**F**,**I**) Quantification of proliferating CD4^+^ (**F**) and CD8^+^ (I) T-cells based on CFSE dilution. Statistical analysis was performed using one-way ANOVA. (**G**,**J**) Representative bright-field images of PBMCs co-cultured with WT-iMSCs (**G**) or dKO-iMSCs (**J**). Data are presented as mean ± SD (*n* = 3). ns, not significant, *** *p* < 0.001, **** *p* < 0.0001.

Flow cytometry was performed to determine whether the effects of double-knockout were maintained following differentiation into iMSCs. Under basal conditions, HLA-ABC was barely detectable on the cell surface of dKO-iMSCs compared with WT-iMSCs (Figure 3A). However, both WT-iMSCs and dKO-iMSCs exhibited only minimal basal expression of HLA-DR with no significant difference between the two groups (Figure 3B). Following IFN-γ stimulation, HLA-ABC expression was increased in WT-iMSCs, whereas HLA-ABC remained undetectable in dKO-iMSCs (Figure 3A). Similarly, IFN-γ stimulation substantially increased HLA-DR expression in WT-iMSCs, while no apparent HLA-DR expression was detected in dKO-iMSCs (Figure 3B). These results demonstrate that B2M and CIITA double knockout stably eliminate expression of HLA class I and class II molecules on the cell surface in dKO-iMSCs, even under inflammatory conditions.

To determine whether B2M and CIITA double knockout affected the intrinsic immunomodulatory activity of iMSCs, WT-iMSCs and dKO-iMSCs were separately co-cultured with human peripheral blood mononuclear cells (PBMCs), with PHA-L stimulation used to establish an activated PBMC model. Cell proliferation was assessed by CFSE dilution to compare the inhibitory effects of WT-iMSCs and dKO-iMSCs on activated immune cells. PHA-L stimulation markedly induced PBMC proliferation, while both WT-iMSCs and dKO-iMSCs significantly suppressed the proliferation of PHA-L-stimulated PBMCs, with no significant difference in inhibitory activity between the two groups (Figure 3C,D).

To further evaluate the immunomodulatory effects of the two types of iMSCs on T-cell subsets, the proliferation of CD4^+^ and CD8^+^ T-cell subsets was analyzed. Both WT-iMSCs and dKO-iMSCs significantly suppressed the proliferation of activated CD4^+^ T-cells, with comparable inhibitory effects between the two groups (Figure 3E,F). Similarly, both WT-iMSCs and dKO-iMSCs significantly inhibited the proliferation of activated CD8^+^ T-cells, with no significant difference between the two groups (Figure 3H,I). Representative bright-field images showed the cellular morphology and culture characteristics of the PBMC and iMSC co-culture systems (Figure 3G,J).

These results demonstrate that B2M and CIITA double knockout did not influence the ability of iMSCs to suppress the proliferation of activated PBMCs or T-cells. Thus, dKO-iMSCs retained the intrinsic immunomodulatory activity of MSCs despite the substantial reduction in HLA expression, providing a functional basis for subsequent evaluation of their therapeutic potential in vivo.

### 3.4. dKO-iMSCs Alleviate Cartilage Degeneration in Mouse Models of OA

**Figure 4:**
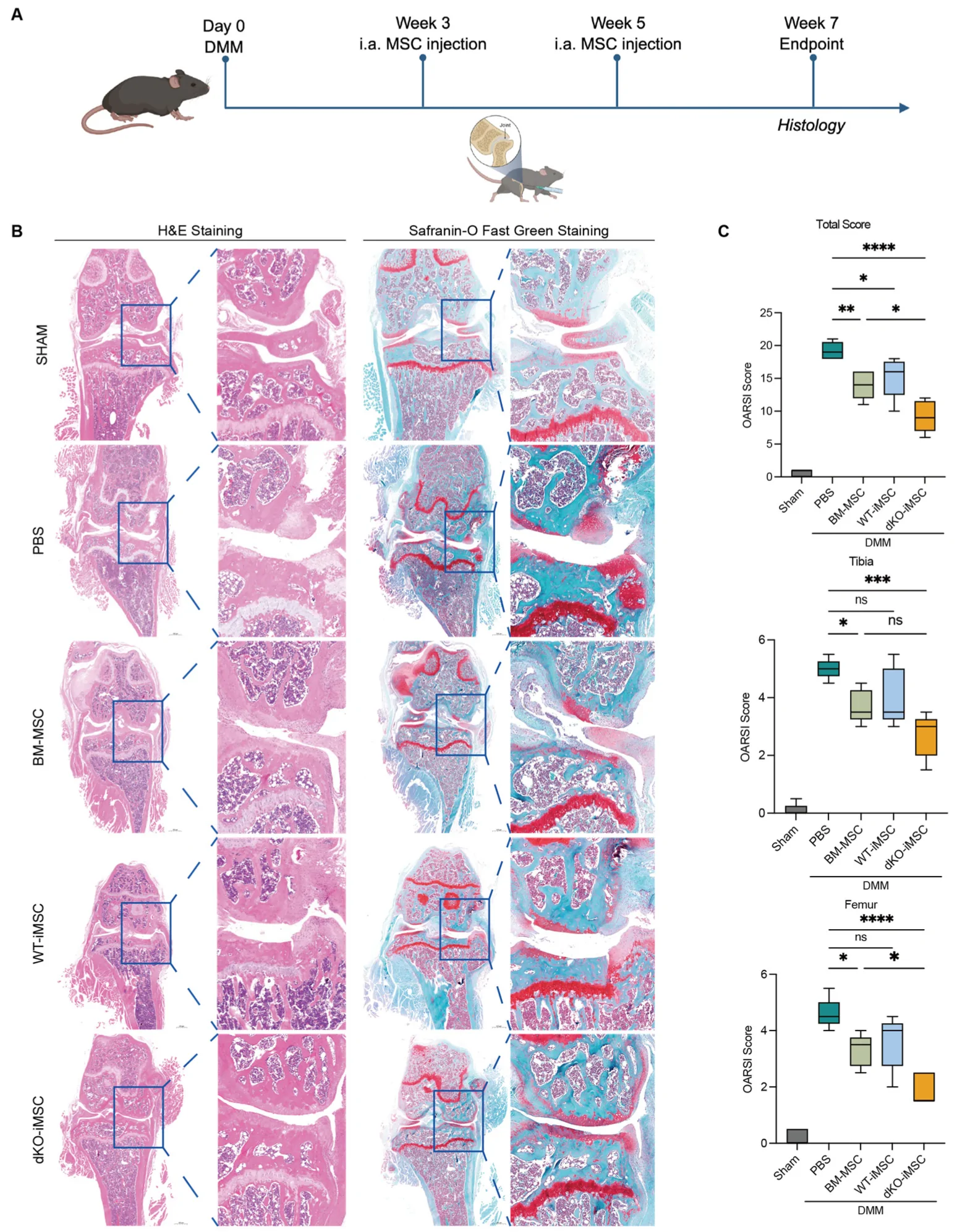
Intra-articular injection of dKO-iMSCs alleviates cartilage degeneration in an OA mouse model. (**A**) Experimental timeline of the OA model and cell treatment. DMM surgery was performed at week 0, followed by intra-articular administration of PBS, BM-MSCs, WT-iMSCs, or dKO-iMSCs at weeks 3 and 5. Mice were euthanized at week 7 for histological evaluation. (**B**) Representative H&E- and Safranin O-Fast Green-stained sections of knee joints from the sham, PBS, BM-MSC, WT-iMSC, and dKO-iMSC groups. Dashed boxes indicate the regions shown at higher magnification. (**C**) Quantification of OARSI scores, including total, tibial, and femoral compartment scores, in the five groups (*n* = 5 mice per group). Scores were independently assessed by two observers blinded to the treatment groups. Box plots show the median and interquartile range, with whiskers indicating the minimum and maximum values. Statistical analysis was performed using one-way ANOVA. ns, not significant, * *p* < 0.05, ** *p* < 0.01, *** *p* < 0.001, **** *p* < 0.0001.

To further evaluate the therapeutic efficacy and potential translational value of genetically engineered iMSCs in vivo, we established a surgically induced mouse model of osteoarthritis by DMM. BM-MSCs, WT-iMSCs, or dKO-iMSCs were administered by intra-articular injection at weeks 3 and 5 after DMM surgery, and the animals were sacrificed at week 7 for endpoint evaluation. The effects of each cell treatment on cartilage degeneration and systemic safety were subsequently assessed (Figure 4A).

Histological analysis revealed obvious degenerative changes in the articular cartilage of PBS group compared with the sham group, including cartilage thinning, surface irregularity and erosion, extensive loss of proteoglycan matrix, and disorganization or focal loss of chondrocytes, consistent with the characteristic pathological features of DMM induced OA. Cell treatment partially attenuated these pathological changes. BM-MSC and WT-iMSC treatment showed reduced cartilage surface damage and partial preservation of proteoglycan-rich matrix in joints, whereas dKO-iMSC treatment exhibited better preservation of cartilage structural integrity, greater residual cartilage thickness, improved extracellular matrix preservation, and a relatively organized chondrocyte arrangement in joints (Figure 4B). OARSI scores were independently assessed by two blinded observers. Compared with the PBS group, both BM-MSC and WT-iMSC treatment significantly reduced the total OARSI score. Notably, dKO-iMSC treatment achieved a greater reduction, with the total OARSI score significantly lower than that of the WT-iMSC and BM-MSC (*p* < 0.05) groups. The femoral score of dKO-iMSC group was lower than WT-iMSC and BM-MSC (*p* < 0.05) groups, and dKO-iMSC-treated mice also exhibited the lowest tibial scores (Figure 4C). These results indicate that dKO-iMSCs retained the most effective cartilage protection among the three cell treatments.

The safety of the cell treatments in vivo was further evaluated by measuring the weights of the heart, liver, spleen, lungs, and kidneys at the experimental endpoint. No significant differences in organ weights were observed between any of the cell treated groups and the PBS or sham control group. Moreover, no apparent organ enlargement, atrophy, or gross morphological abnormalities were detected. Consistent with these findings, H&E staining revealed no evident tissue damage or pathological abnormalities in the major organs examined. These results indicate that intra-articular administration of BM-MSCs, WT-iMSCs, or dKO-iMSCs did not result in detectable abnormalities in organ weights (Supplemental Figure S1).

dKO-iMSCs exhibited superior therapeutic efficacy compared with WT-iMSCs and BM-MSCs in mice OA models. No evident systemic toxicity was observed following dKO-iMSC treatment in this study. These findings provide in vivo evidence supporting further evaluation of dKO-iMSCs as a potential cell source for allogeneic OA therapy.

### 3.5. Single-Cell Transcriptomics Reveals Enhanced ECM-Related Communication and Reduced Inflammation in IFN-γ-Stimulated dKO-iMSCs

**Figure 5:**
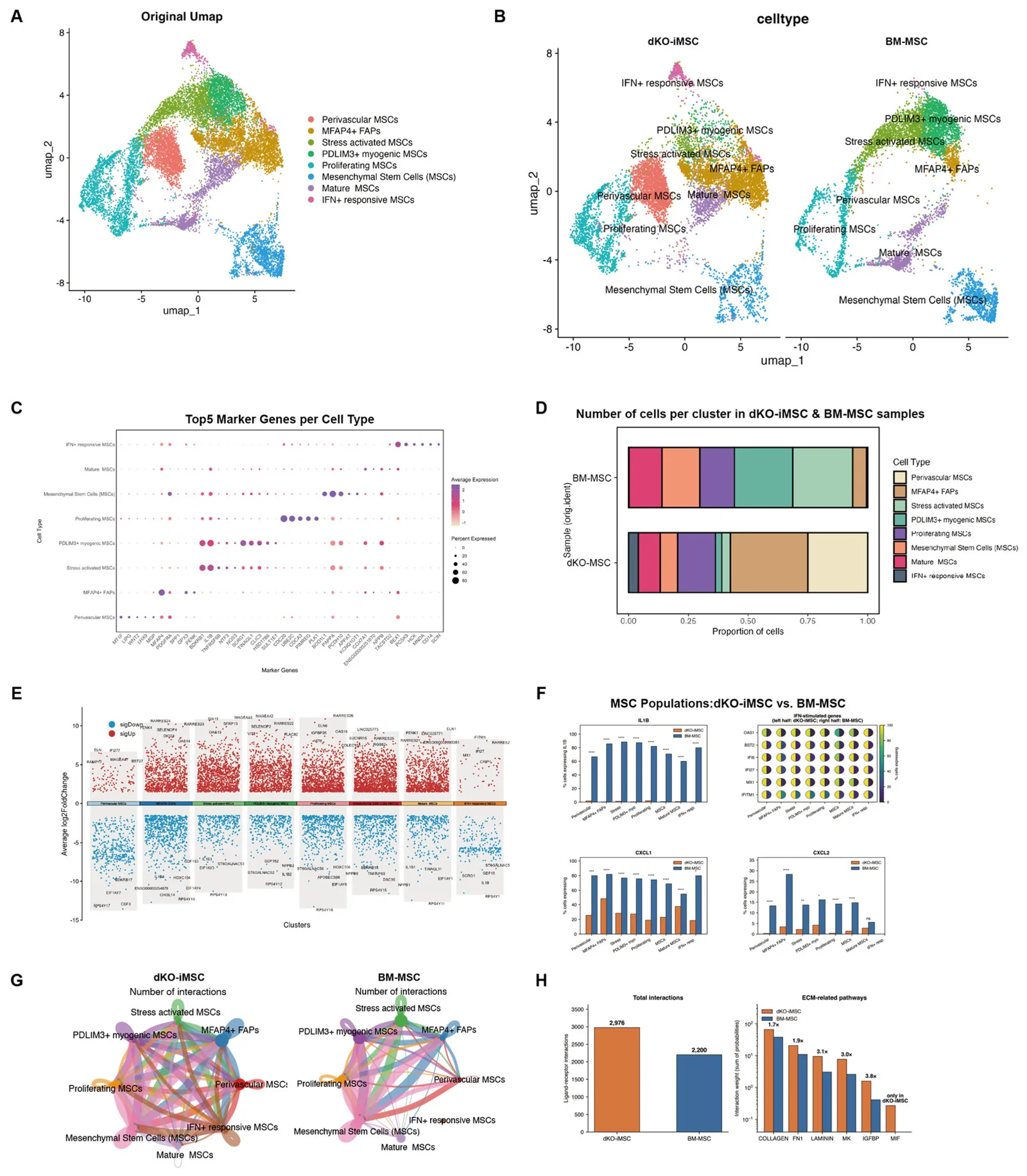
Single-cell transcriptomic profiling of IFN-γ-stimulated BM-MSCs and dKO-iMSCs. (**A**) UMAP projection showing the eight MSC subclusters identified in IFN-γ-stimulated BM-MSCs and dKO-iMSCs. (**B**) UMAP projections showing the distribution of MSC subclusters in dKO-iMSC (left) and BM-MSC (right) groups. (**C**) Dot plot showing the top five marker genes for each MSC subcluster. Dot size indicates the fraction of cells expressing the gene, and color indicates scaled average expression. (**D**) Relative proportions of the eight MSC subclusters in the dKO-iMSC and BM-MSC groups. (**E**) Differentially expressed genes between dKO-iMSCs and BM-MSCs within each MSC subcluster. Red indicates genes upregulated in dKO-iMSCs, and blue indicates genes downregulated in dKO-iMSCs (log_2_ fold change ≥ 1.5, *p* ≤ 0.05; two-sided Wilcoxon rank-sum test with Bonferroni correction). (**F**) Expression frequencies of IFN-stimulated genes (left half, dKO-iMSCs; right half, BM-MSCs), IL1B, CXCL1, and CXCL2 across the eight subclusters (a gene was considered expressed when at least one UMI was detected). (**G**) CellChat network plots of inferred interactions among the eight subclusters in each cell type. Node size indicates the number of interactions. (**H**) The total numbers of interactions were 2976 and 2200 in the dKO-iMSC and BM-MSC groups, respectively. ECM-associated pathways showed increased interaction weights in the dKO-iMSC group, including COLLAGEN (1.7-fold), FN1 (1.9-fold), LAMININ (3.1-fold), MK (3.0-fold), and IGFBP (3.8-fold), whereas MIF-mediated signaling was detected only in the dKO-iMSC group. ns, not significant, * *p* < 0.05, ** *p* < 0.01, *** *p* < 0.001, **** *p* < 0.0001.

To characterize the transcriptional response of the engineered cells to inflammatory stimulation, the same preparations of BM-MSCs and dKO-iMSCs used for intra-articular injection were stimulated with IFN-γ and subjected to single-cell RNA sequencing. Unsupervised clustering with canonical marker gene annotation identified eight MSC subclusters, including MSCs, mature MSCs, perivascular MSCs, MFAP4+ fibroblast-adipocyte progenitor-like cells (MFAP4+ FAPs), proliferating MSCs, stress-activated MSCs, PDLIM3+ myogenic MSCs, and IFN+ responsive MSCs (Figure 5A,B). Each subcluster displayed a distinct marker gene signature (Figure 5C).

All eight subclusters were detected in both groups, but their relative abundances differed markedly (Figure 5D). The dKO-iMSC group was dominated by MFAP4+ FAPs (33.4%) and perivascular MSCs (25.6%), followed by proliferating MSCs (15.9%). In contrast, stress-activated MSCs (25.5%) and PDLIM3+ myogenic MSCs (24.7%) were the most abundant subclusters in the BM-MSC group. Perivascular MSCs (25.6% vs. <1%) and IFN-responsive MSCs (3.9% vs. 0.3%) were detected almost exclusively in the dKO-iMSC group.

At the transcriptional level, a prominent difference between the two groups was the contrasting expression of interferon-stimulated genes (ISGs) and IL1B. In the MSC subcluster, ISGs including IFITM1 (99.2% vs. 19.6%), MX1 (90.2% vs. 4.7%), IFI27 (93.4% vs. 12.3%), IFI6 (84.8% vs. 9.5%), BST2 (84.9% vs. 8.4%), and OAS1 (66.8% vs. 1.0%) were expressed by the majority of the dKO-iMSC group but only a minority of the BM-MSC group. In contrast, IL1B was detected in 70.7% of BM-MSCs but only 0.6% of cells in the dKO-iMSC group. This difference was consistently observed across the eight subclusters, with IL1B-positive cells accounting for 60–88% of cells in the BM-MSC group but less than 2% of cells in the dKO-iMSC group. The pro-inflammatory chemokines CXCL1 and CXCL2 were also reduced in the dKO-iMSC group. For each of the eight MSC subclusters, differentially expressed genes (DEGs) between the dKO-iMSC and BM-MSC groups were identified separately. Genes upregulated in the dKO-iMSC group with a log2 fold change ≥ 1.5 and *p* ≤ 0.05 were subsequently subjected to Gene Ontology (GO) enrichment analysis. In IFN+ responsive MSCs, the upregulated genes were enriched in IFN related biological processes, including negative regulation of viral genome replication. In contrast, ECM-associated GO terms, including extracellular matrix constituent and lubricant activity, were enriched in proliferating MSCs, while extracellular matrix binding and extracellular matrix structural constituent were enriched in perivascular MSCs (Figure 5E,F).

Consistent with these ECM-associated transcriptional features, CellChat analysis of the eight subclusters (Figure 5G,H) detected more ligand–receptor interactions in the dKO-iMSC group (2976 vs. 2200 interactions in the BM-MSC group). ECM-related pathways were enhanced in the dKO-iMSC group, including COLLAGEN (1.7-fold), FN1 (1.9-fold), LAMININ (3.1-fold), MK (3.0-fold), and IGFBP (3.8-fold), and MIF-mediated signaling was detected only in the dKO-iMSC group.

Together, these data indicate that upon IFN-γ stimulation, the dKO-iMSC group maintained a low-IL1B, high-ISG transcriptional state with enhanced ECM-related cell–cell communication. These findings suggest that HLA-engineered iMSCs are less prone to adopt a pro-inflammatory transcriptional program under inflammatory stimulation, which may contribute to their therapeutic advantage observed in vivo.

## 4. Discussion

Allogeneic MSC transplantation is an attractive strategy for OA cell therapy, but its broader application remains limited by concerns regarding host immune recognition. Inflammatory cytokines within the joint microenvironment can increase HLA expression on MSCs, potentially enhancing their recognition by the recipient immune system [18]. In this study, we combined HLA-targeted genome engineering with iPSC platforms to generate B2M/CIITA double-knockout iMSCs. These cells showed sustained loss of HLA class I and class II surface expression under both basal and IFN-γ-stimulated conditions, while retaining MSC identity, long-term proliferative capacity, and immunomodulatory activity. In mouse DMM models, dKO-iMSCs provided more cartilage protection than the other cell treatments without evident toxicity. Single-cell transcriptomics of IFN-γ-stimulated cells further revealed that dKO-iMSCs were characterized by reduced IL1B expression, an enhanced interferon-stimulated gene (ISG) signature, and increased ECM-associated cell–cell communication, indicating a less pro-inflammatory response to inflammatory stimulation. These findings support that substantial reduction in HLA expression can be achieved while preserving the characteristic biological properties of iMSCs and enhancing their therapeutic performance in experimental OA.

Genetic disruption of HLA-related pathways has previously been explored in pluripotent stem cells and their differentiated derivatives [16,26,30,31]. Our findings extend these observations by demonstrating that simultaneous disruption of B2M and CIITA can maintain reduced HLA class I and class II surface expression following differentiation into iMSCs and under inflammatory stimulation. Importantly, HLA disruption did not eliminate the cellular response to inflammatory signaling. After IFN-γ stimulation in vitro, dKO-iMSCs retained a pronounced ISG signature, indicating that suppression of surface HLA expression does not prevent these cells from responding transcriptionally to inflammatory signaling. This observation is consistent with the concept that MSCs exhibit immunomodulatory or immune-evasive properties rather than complete immune privilege [20].

Preservation of MSC biological function is as important as the reduction in immunogenicity [32]. dKO-iMSCs fulfilled MSC phenotypic criteria and retained trilineage differentiation potential. dKO-iMSCs suppressed CD4+ T-cell and CD8+ T-cell proliferation to a degree comparable to WT-iMSCs. These findings indicate that B2M/CIITA double knockout did not impair the intrinsic immunomodulatory activity of iMSCs. In addition, dKO-iMSCs maintained stable proliferation through passage 18, whereas BM-MSCs showed progressive growth arrest after passage 8. This long-term expansion capacity highlights an important advantage of the iPSC platform for MSC manufacturing, as primary tissue-derived MSCs are subject to donor-dependent variability, limited cell availability, and replicative senescence [33]. Importantly, dKO-iMSCs provided more cartilage protection than WT-iMSCs and BM-MSCs in the DMM model. Although the mechanisms underlying this efficacy remain to be fully elucidated, single-cell transcriptomics revealed that IFN-γ-stimulated dKO-iMSCs exhibited markedly reduced IL1B and CXCL1/CXCL2 expression, together with enhanced ISG signatures and ECM-associated cell–cell communication. The enrichment of MFAP4^+^ FAPs and perivascular MSCs further coincided with ECM-associated transcriptional features. Thus, the combination of a renewable iPSC source with targeted HLA engineering may provide complementary advantages for developing more standardized allogeneic MSC products for OA therapy.

Several limitations of the present study should be acknowledged. First, the findings of single-cell transcriptomics identified reduced IL1B expression and enhanced ECM-associated communication in dKO-iMSC treatment group, but these observations remain correlative. The functional contribution of these pathways, including the MIF signaling detected exclusively in the dKO-iMSC group, will require further mechanistic investigation. Second, the DMM study was conducted over a relatively short observation period. Longer-term studies will be important to further evaluate the durability of therapeutic effects, biodistribution, and safety, as well as potential dose-dependent effects. Although no apparent abnormalities in major organ weights were observed at the study endpoint, the safety assessment was limited to endpoint evaluation and does not exclude potential delayed or long-term systemic effects. In addition, the relatively small number of animals per group (*n* = 5), together with the multiple-group comparison design, may have limited the statistical power of the OARSI analysis and should therefore be considered when interpreting the histological outcomes. Further studies with longer follow-up and larger sample sizes will be valuable to validate these findings. Third, B2M disruption reduces cell-surface HLA class I expression, so its potential effects on NK cell recognition warrant further investigation. In particular, the potential contribution of NK-cell inhibitory molecules, such as HLA-E and CD47, to the immune compatibility of engineered MSCs should be explored in future studies [34,35,36]. Fourth, genome-wide off-target effects of CRISPR/Cas9 editing were not assessed using unbiased approaches such as whole-genome sequencing or GUIDE-seq. Although the edited clone was validated by Sanger sequencing of the target loci and G-banded karyotyping, these analyses cannot exclude undetected genome-wide off-target alterations. Comprehensive genome-wide genomic analyses will therefore be needed to further evaluate the genetic safety of the engineered cells. Finally, the assessment relevant to immunogenicity in the present study was limited to the complete and sustained loss of surface HLA class I and class II expression. Direct functional assays of allogeneic T-cell activation, cytotoxicity, and immune-mediated clearance were not performed. In addition, the in vivo OA model using immunocompetent C57BL/6 mice represents a xenogeneic setting that does not recapitulate human allogeneic immune compatibility. The model was primarily designed to evaluate the cartilage-protective and tissue-reparative effects of iMSCs, and the immune compatibility and persistence of dKO-iMSCs were not directly assessed in vivo. Future studies incorporating allogeneic co-culture and immune-mediated clearance assays in vitro, as well as humanized immune system mouse models in vivo, will be needed to directly evaluate the immune compatibility and persistence of dKO-iMSCs [37,38].

The combination of stable HLA silencing and the scalability of the iPSC platform provide a potential route toward more standardized, off-the-shelf allogeneic MSC products for OA and other regenerative medicine applications. Further studies addressing in vivo immune evasion, long-term safety, and the mechanisms underlying the enhanced therapeutic effects of dKO-iMSCs will be important for advancing this approach toward clinical translation. Overall, this study demonstrates that B2M/CIITA double knockout substantially reduces HLA class I and class II expression while preserving the characteristic biological and immunomodulatory properties of iMSCs and enhancing their therapeutic effects in a mouse OA model. These findings provide a foundation for further development of HLA-engineered iMSCs as a potential allogeneic cell therapy for OA.

## 5. Conclusions

B2M/CIITA double knockout iPSC-derived MSCs preserve MSC identity, trilineage differentiation potential, long-term expansion capacity, and immunomodulatory activity while exhibiting no detectable HLA class I or class II expression. In the OA mouse model, dKO-iMSCs provided superior therapeutic efficacy among the tested cell treatments, as evidenced by improved OARSI scores, without evident systemic toxicity. Single-cell RNA sequencing further showed that dKO-iMSCs retained a high-ISG, low-IL1B transcriptional state under inflammatory conditions and exhibited enhanced extracellular matrix-associated cell–cell communication, consistent with a less pro-inflammatory and more matrix-supportive phenotype.

## Data Availability

The single-cell RNA-sequencing data generated in this study have been deposited in the NCBI Sequence Read Archive (SRA) under BioProject accession number PRJNA1532881. All other data supporting the findings of this study are available from the corresponding author upon reasonable request.

## Supplementary Materials

The following supporting information can be downloaded at: https://www.mdpi.com/article/10.3390/cells15191777/s1, Figure S1: Weights of major organs in mice after intra-articular administration of iMSCs.
